# Crystal structure of 3′-(1*H*-indole-3-carbon­yl)-1′-methyl-2-oxo-4′-(4-oxo-4*H*-chromen-3-yl)spiro­[indoline-3,2′-pyrrolidine]-3′-carbo­nitrile

**DOI:** 10.1107/S2056989015020174

**Published:** 2015-10-31

**Authors:** M. P. Savithri, R. Raja, D. Kathirvelan, B. S. R. Reddy, A. SubbiahPandi

**Affiliations:** a Department of Physics, Queen Mary’s College (Autonomous), Chennai 600 004, India; b Department of Physics, Presidency College (Autonomous), Chennai 600 005, India; c Industrial Chemistry Laboratory, Central Leather Research Institute, Adyar, Chennai 600 020, India

**Keywords:** crystal structure, indole, pyrrolidine, chromen, spiro, carbo­nitrile, hydrogen bonding

## Abstract

In the title compound, C_31_H_22_N_4_O_4_, the pyrrolidine ring adopts a twist conformation on the N—CH_2_ bond. The indolin-2-one and the 1*H*-indole rings are nearly planar (r.m.s. deviations = 0.06 and 0.011 Å, respectively) and are inclined to one another by 34.19 (9)°. The chromene ring system is also nearly planar (r.m.s. deviation = 0.029 Å). It is almost normal to the 1*H*-indole ring system, with a dihedral angle of 88.71 (8)°, and is inclined to the indolin-2-one ring system by 72.76 (8)°. In the crystal, mol­ecules are linked *via* N—H⋯O hydrogen bonds, forming slabs parallel to (10-1). The slabs are linked by C—H⋯O hydrogen bonds, forming a three-dimensional structure.

## Related literature   

For the biological activities of indole derivatives, see: Macor *et al.* (1992 ▸); Andreani *et al.* (2001 ▸); Quetin-Leclercq (1994 ▸); Mukhopadhyay *et al.* (1981 ▸); Singh *et al.* (2000 ▸). For the structure of a very similar compound, see: Ramesh *et al.* (2009 ▸).
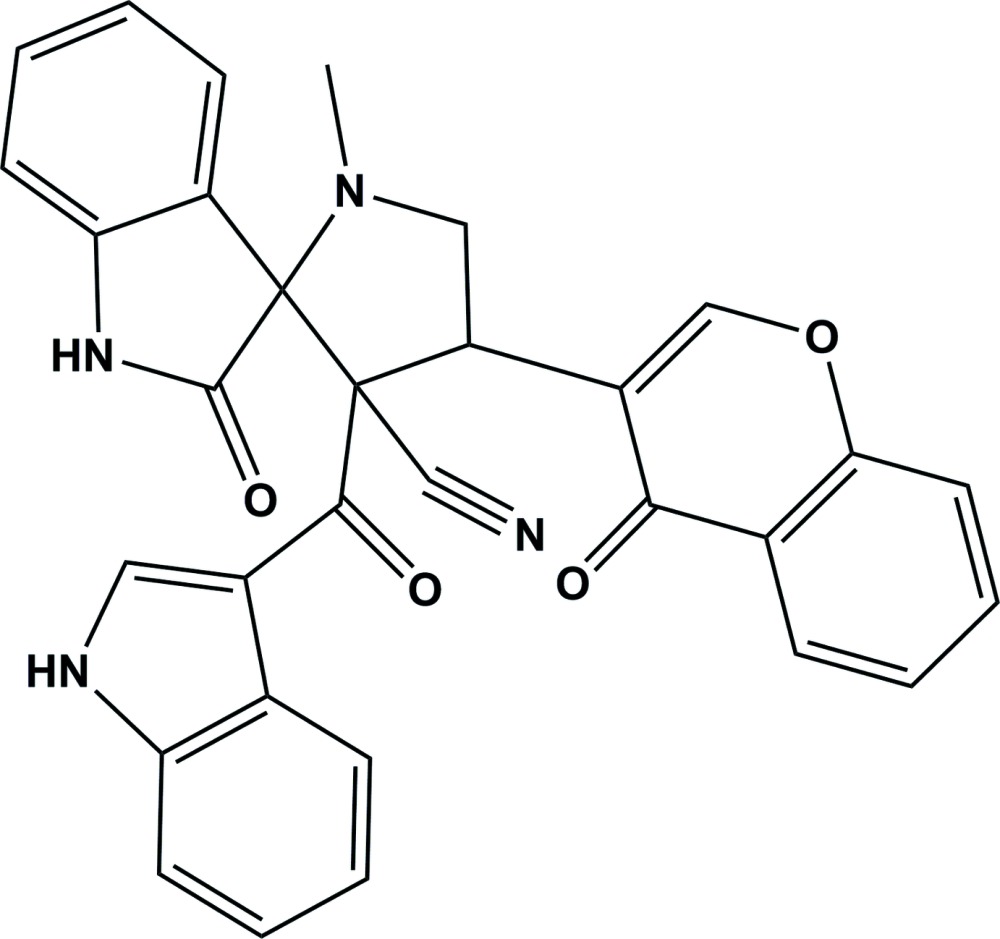


## Experimental   

### Crystal data  

C_31_H_22_N_4_O_4_
*M* *_r_* = 514.53
Monoclinic, 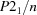

*a* = 13.0401 (5) Å
*b* = 14.9139 (6) Å
*c* = 13.7161 (5) Å
β = 112.603 (2)°
*V* = 2462.60 (16) Å^3^
*Z* = 4
Mo *K*α radiation
μ = 0.09 mm^−1^
*T* = 293 K
0.35 × 0.30 × 0.30 mm

### Data collection  

Bruker Kappa APEXII CCD diffractometer
Absorption correction: multi-scan (*SADABS*; Bruker, 2004 ▸) *T*_min_ = 0.969, *T*_max_ = 0.974
16591 measured reflections
4341 independent reflections
3234 reflections with *I* > 2σ(*I*)
*R*_int_ = 0.030

### Refinement  

*R*[*F* ^2^ > 2σ(*F* ^2^)] = 0.049
*wR*(*F* ^2^) = 0.132
*S* = 1.04
4329 reflections
353 parameters
H-atom parameters constrained
Δρ_max_ = 0.29 e Å^−3^
Δρ_min_ = −0.18 e Å^−3^

### 

Data collection: *APEX2* (Bruker, 2004 ▸); cell refinement: *APEX2* and *SAINT* (Bruker, 2004 ▸); data reduction: *SAINT* and *XPREP* (Bruker, 2004 ▸); program(s) used to solve structure: *SHELXS97* (Sheldrick, 2008 ▸); program(s) used to refine structure: *SHELXL97* (Sheldrick, 2008 ▸); molecular graphics: *PLATON* (Spek, 2009 ▸); software used to prepare material for publication: *SHELXL97* and *PLATON*.

## Supplementary Material

Crystal structure: contains datablock(s) global, I. DOI: 10.1107/S2056989015020174/su5215sup1.cif

Structure factors: contains datablock(s) I. DOI: 10.1107/S2056989015020174/su5215Isup2.hkl

Supporting information file. DOI: 10.1107/S2056989015020174/su5215Isup3.cml

**Fig. 1.**
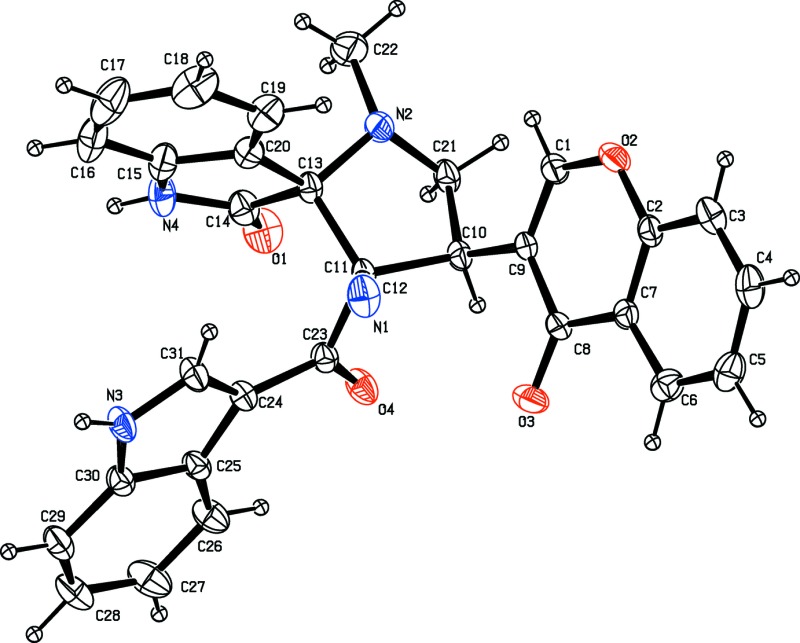
The molecular structure of the title compound, with atom labelling. Displacement ellipsoids are drawn at the 30% probability level.

**Fig. 2.**
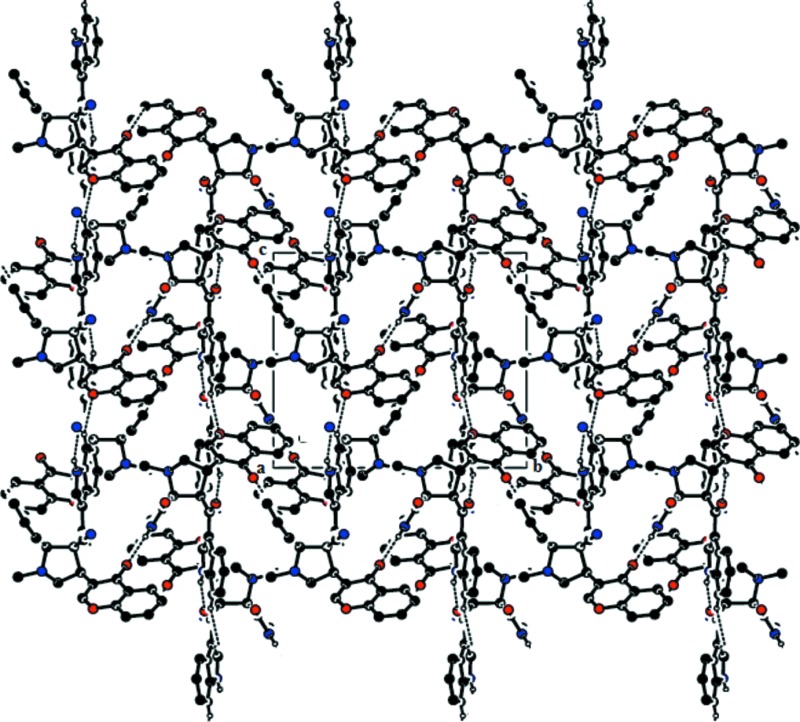
A view along the b axis of the crystal packing of the title compound. The hydrogen bonds are shown as dashed lines (see Table 1).

. DOI: 10.1107/S2056989015020174/su5215fig1.tif
The mol­ecular structure of the title compound, with atom labelling. Displacement ellipsoids are drawn at the 30% probability level.

b . DOI: 10.1107/S2056989015020174/su5215fig2.tif
A view along the *b* axis of the crystal packing of the title compound. The hydrogen bonds are shown as dashed lines (see Table 1).

CCDC reference: 1433172

Additional supporting information:  crystallographic information; 3D view; checkCIF report

## Figures and Tables

**Table 1:** Hydrogen-bond geometry (, )

*D*H*A*	*D*H	H*A*	*D* *A*	*D*H*A*
N3H3*A*O4^i^	0.86	2.14	2.967(3)	161
N4H4*A*O3^ii^	0.86	2.06	2.866(3)	156
C29H29O2^iii^	0.93	2.59	3.267(3)	131

Symmetry codes: (i) 
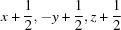
; (ii) 
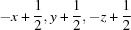
; (iii) 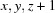.

## supplementary crystallographic information

### S1. Structural commentary

\ The chemistry of indole has been of increasing inter­est, since several compounds of this type possess diverse biological activities (Macor *et al.*, 1992). These derivatives exhibit anti­bacterial, anti­fungal (Singh *et al.*, 2000) and anti­tumour activities (Andreani *et al.*, 2001). Some of the indole alkaloids extracted from plants possess inter­esting cytotoxic and anti­parasitic properties (Quetin-Leclercq, 1994; Mukhopadhyay *et al.*, 1981).

The geometric parameters of the title molecule (Fig. 1) agree well with those reported for a similar compound, 1'-methyl-2,2''-dioxoindoline-3-spiro-2'-pyrrolidine-3'-spiro-3''-indoline-\ 4',4'-dicarbo­nitrile (Ramesh *et al.*, 2009). The pyrrolidine ring (N2/C10/C11/C13/C21) adopts a twist conformation on bond N2—C21: puckering parameters and the asymmetry parameters for this ring are q_2_ = 0.411 (2) Å, φ_2_ = 160.3 (3)° nd ΔCs(C11) = 1.3 (2)°. Both indole rings, (N3/24–31) and (N4/C13—C20), are planar [maximum deviations of 0.013 Å for C27 and 0.080 (1) Å for C13 in the two rings] and are oriented at a dihedral angle of 34.19 (9)°. The sum of the bond angles around atom N2 of the central pyrrolidine ring is 337° as expected for *sp*^3^ hybridization.

In the crystal, molecules are linked by N—H···O hydrogen bonds forming slabs parallel to (101). The slabs are linked via C—H···O hydrogen bonds forming a three-dimensional structure (Table 1 and Fig. 2).

### S2. Synthesis and crystallization

A mixture of isatin2a-f (1.0 mmol), sarcosine3 (1.1 mmol) and (*E*)-2-(1*H*-indole -3-carbonyl)-3-(4-oxo-4*H*-chromen-3-yl)acrylo­nitrile 1 (1.2 mmol) in methanol was stirred at room temperature for 120 min. The solid precipitated during the reaction mixture was filtered and dried under vacuum to obtain spiro­oxindoles5a-f in crude form. The resulting crude product was purified by flash column chromatography (mesh 100–200) using hexane/EtOAC (7:3). The solid single product was finally recrystallized from ethanol, giving title compound in good yield as colourless block-like crystals.

### S3. Refinement

Crystal data, data collection and structure refinement details are summarized in Table 2. N and C-bound H atoms were positioned geometrically (N—H = 0.86 Å, C–H = 0.93–0.98 Å) and allowed to ride on their parent atoms, with *U*_iso_(H) = 1.5*U*_eq_(C) for methyl H atoms and 1.2*U*_eq_(N,C) for other H atoms.

### Crystal data

**Table:**

C_31_H_22_N_4_O_4_	*F*(000) = 1072
*M**_r_* = 514.53	*D*_x_ = 1.388 Mg m^−^^3^
Monoclinic, *P*2_1_/*n*	Mo *K*α radiation, λ = 0.71073 Å
Hall symbol: -P 2ybn	Cell parameters from 4241 reflections
*a* = 13.0401 (5) Å	θ = 1.8–25.0°
*b* = 14.9139 (6) Å	µ = 0.09 mm^−^^1^
*c* = 13.7161 (5) Å	*T* = 293 K
β = 112.603 (2)°	Block, colourless
*V* = 2462.60 (16) Å^3^	0.35 × 0.30 × 0.30 mm
*Z* = 4	

### Data collection

**Table:**

Bruker Kappa APEXII CCD diffractometer	4341 independent reflections
Radiation source: fine-focus sealed tube	3234 reflections with *I* > 2σ(*I*)
Graphite monochromator	*R*_int_ = 0.030
ω and φ scans	θ_max_ = 25.0°, θ_min_ = 1.8°
Absorption correction: multi-scan (*SADABS*; Bruker, 2004)	*h* = −14→15
*T*_min_ = 0.969, *T*_max_ = 0.974	*k* = −17→17
16591 measured reflections	*l* = −16→16

### Refinement

**Table:**

Refinement on *F*^2^	Primary atom site location: structure-invariant direct methods
Least-squares matrix: full	Secondary atom site location: difference Fourier map
*R*[*F*^2^ > 2σ(*F*^2^)] = 0.049	Hydrogen site location: inferred from neighbouring sites
*wR*(*F*^2^) = 0.132	H-atom parameters constrained
*S* = 1.04	*w* = 1/[σ^2^(*F*_o_^2^) + (0.0575*P*)^2^ + 1.2814*P*] where *P* = (*F*_o_^2^ + 2*F*_c_^2^)/3
4329 reflections	(Δ/σ)_max_ < 0.001
353 parameters	Δρ_max_ = 0.29 e Å^−^^3^
0 restraints	Δρ_min_ = −0.18 e Å^−^^3^

### Special details

**Table:**

Geometry. All e.s.d.'s (except the e.s.d. in the dihedral angle between two l.s. planes) are estimated using the full covariance matrix. The cell e.s.d.'s are taken into account individually in the estimation of e.s.d.'s in distances, angles and torsion angles; correlations between e.s.d.'s in cell parameters are only used when they are defined by crystal symmetry. An approximate (isotropic) treatment of cell e.s.d.'s is used for estimating e.s.d.'s involving l.s. planes.
Refinement. Refinement of *F*^2^ against ALL reflections. The weighted *R*-factor *wR* and goodness of fit *S* are based on *F*^2^, conventional *R*-factors *R* are based on *F*, with *F* set to zero for negative *F*^2^. The threshold expression of *F*^2^ > σ(*F*^2^) is used only for calculating *R*-factors(gt) *etc*. and is not relevant to the choice of reflections for refinement. *R*-factors based on *F*^2^ are statistically about twice as large as those based on *F*, and *R*- factors based on ALL data will be even larger.

### Fractional atomic coordinates and isotropic or equivalent isotropic displacement parameters (Å^2^)

**Table:**

	*x*	*y*	*z*	*U*_iso_*/*U*_eq_	
C1	0.2880 (2)	0.25426 (16)	−0.10673 (17)	0.0378 (6)	
H1	0.2737	0.3152	−0.1193	0.045*	
C2	0.36735 (18)	0.12268 (16)	−0.14083 (15)	0.0353 (5)	
C3	0.4277 (2)	0.08350 (19)	−0.19416 (18)	0.0467 (6)	
H3	0.4542	0.1178	−0.2361	0.056*	
C4	0.4468 (2)	−0.00645 (19)	−0.18327 (19)	0.0517 (7)	
H4	0.4867	−0.0338	−0.2186	0.062*	
C5	0.4078 (2)	−0.05821 (18)	−0.1203 (2)	0.0498 (7)	
H5	0.4201	−0.1198	−0.1153	0.060*	
C6	0.35133 (19)	−0.01845 (17)	−0.06596 (19)	0.0427 (6)	
H6	0.3269	−0.0529	−0.0226	0.051*	
C7	0.33001 (17)	0.07404 (15)	−0.07526 (15)	0.0325 (5)	
C8	0.27094 (18)	0.11941 (15)	−0.01806 (15)	0.0340 (5)	
C9	0.24912 (18)	0.21424 (15)	−0.04083 (15)	0.0307 (5)	
C10	0.18396 (18)	0.26261 (15)	0.01285 (15)	0.0321 (5)	
H10	0.1243	0.2227	0.0130	0.039*	
C11	0.25597 (17)	0.28881 (15)	0.13121 (15)	0.0298 (5)	
C12	0.3687 (2)	0.25089 (15)	0.16436 (16)	0.0338 (5)	
C13	0.26070 (17)	0.39617 (15)	0.13015 (15)	0.0315 (5)	
C14	0.1881 (2)	0.43664 (17)	0.18886 (18)	0.0424 (6)	
C15	0.3657 (2)	0.48427 (17)	0.27711 (19)	0.0464 (6)	
C16	0.4568 (3)	0.52655 (19)	0.3503 (2)	0.0641 (9)	
H16	0.4530	0.5548	0.4093	0.077*	
C17	0.5530 (3)	0.5251 (2)	0.3323 (3)	0.0734 (10)	
H17	0.6159	0.5529	0.3804	0.088*	
C18	0.5596 (2)	0.4835 (2)	0.2449 (3)	0.0683 (9)	
H18	0.6256	0.4856	0.2338	0.082*	
C19	0.4675 (2)	0.43842 (19)	0.1731 (2)	0.0507 (7)	
H19	0.4715	0.4095	0.1146	0.061*	
C20	0.37124 (19)	0.43801 (16)	0.19138 (17)	0.0378 (6)	
C21	0.13173 (19)	0.35150 (16)	−0.03531 (16)	0.0382 (6)	
H21A	0.1129	0.3522	−0.1110	0.046*	
H21B	0.0653	0.3635	−0.0217	0.046*	
C22	0.1867 (2)	0.50981 (18)	−0.0085 (2)	0.0581 (8)	
H22A	0.1694	0.5195	−0.0823	0.087*	
H22B	0.2473	0.5479	0.0325	0.087*	
H22C	0.1227	0.5236	0.0073	0.087*	
C23	0.19977 (18)	0.25368 (15)	0.20664 (16)	0.0331 (5)	
C24	0.25979 (18)	0.25638 (15)	0.32050 (16)	0.0339 (5)	
C25	0.21092 (18)	0.23643 (15)	0.39638 (16)	0.0338 (5)	
C26	0.1073 (2)	0.20842 (18)	0.39024 (19)	0.0472 (6)	
H26	0.0500	0.1987	0.3251	0.057*	
C27	0.0911 (2)	0.1953 (2)	0.4831 (2)	0.0603 (8)	
H27	0.0222	0.1757	0.4799	0.072*	
C28	0.1754 (2)	0.2107 (2)	0.5811 (2)	0.0555 (7)	
H28	0.1615	0.2020	0.6421	0.067*	
C29	0.2784 (2)	0.23842 (17)	0.58952 (18)	0.0456 (6)	
H29	0.3350	0.2487	0.6549	0.055*	
C30	0.29469 (19)	0.25050 (16)	0.49630 (16)	0.0359 (5)	
C31	0.36911 (19)	0.27949 (17)	0.37844 (17)	0.0412 (6)	
H31	0.4208	0.2950	0.3499	0.049*	
N1	0.45588 (18)	0.22200 (15)	0.18987 (16)	0.0502 (6)	
N2	0.21796 (16)	0.41585 (13)	0.01775 (13)	0.0390 (5)	
N3	0.38947 (16)	0.27611 (14)	0.48210 (14)	0.0430 (5)	
H3A	0.4522	0.2881	0.5320	0.052*	
N4	0.25757 (18)	0.48177 (15)	0.27375 (16)	0.0521 (6)	
H4A	0.2375	0.5062	0.3204	0.063*	
O1	0.08831 (15)	0.42855 (14)	0.16214 (15)	0.0609 (5)	
O2	0.34652 (14)	0.21307 (11)	−0.15668 (12)	0.0435 (4)	
O3	0.24130 (15)	0.08041 (11)	0.04650 (13)	0.0511 (5)	
O4	0.10336 (13)	0.22832 (12)	0.16760 (12)	0.0457 (4)	

### Atomic displacement parameters (Å^2^)

**Table:**

	*U*^11^	*U*^22^	*U*^33^	*U*^12^	*U*^13^	*U*^23^
C1	0.0488 (14)	0.0357 (13)	0.0302 (11)	0.0036 (11)	0.0165 (11)	−0.0004 (10)
C2	0.0390 (13)	0.0396 (14)	0.0232 (10)	0.0015 (11)	0.0072 (9)	−0.0036 (9)
C3	0.0508 (15)	0.0603 (18)	0.0311 (12)	0.0063 (13)	0.0180 (11)	−0.0023 (11)
C4	0.0495 (16)	0.0614 (19)	0.0413 (14)	0.0121 (14)	0.0144 (12)	−0.0139 (13)
C5	0.0469 (15)	0.0412 (15)	0.0549 (15)	0.0063 (12)	0.0124 (13)	−0.0107 (12)
C6	0.0378 (14)	0.0402 (15)	0.0454 (14)	−0.0025 (11)	0.0108 (11)	−0.0007 (11)
C7	0.0338 (12)	0.0348 (13)	0.0242 (10)	−0.0015 (10)	0.0060 (9)	−0.0028 (9)
C8	0.0406 (13)	0.0360 (13)	0.0226 (10)	−0.0039 (10)	0.0091 (9)	0.0006 (9)
C9	0.0364 (12)	0.0328 (12)	0.0193 (10)	−0.0038 (10)	0.0069 (9)	−0.0030 (9)
C10	0.0319 (12)	0.0381 (13)	0.0229 (10)	−0.0020 (10)	0.0068 (9)	−0.0032 (9)
C11	0.0288 (11)	0.0360 (13)	0.0213 (10)	0.0038 (10)	0.0061 (9)	−0.0026 (9)
C12	0.0371 (13)	0.0382 (14)	0.0242 (10)	0.0036 (11)	0.0098 (10)	−0.0052 (9)
C13	0.0305 (11)	0.0347 (13)	0.0260 (10)	0.0049 (10)	0.0074 (9)	−0.0026 (9)
C14	0.0425 (15)	0.0451 (15)	0.0397 (13)	0.0098 (12)	0.0160 (11)	−0.0009 (11)
C15	0.0494 (16)	0.0387 (14)	0.0400 (13)	0.0073 (12)	0.0046 (11)	−0.0077 (11)
C16	0.063 (2)	0.0510 (18)	0.0545 (17)	0.0071 (15)	−0.0035 (15)	−0.0207 (13)
C17	0.0541 (19)	0.0541 (19)	0.083 (2)	−0.0019 (15)	−0.0054 (16)	−0.0189 (17)
C18	0.0353 (15)	0.063 (2)	0.095 (2)	−0.0018 (14)	0.0114 (15)	0.0006 (18)
C19	0.0374 (14)	0.0514 (17)	0.0585 (16)	−0.0003 (12)	0.0133 (12)	−0.0029 (13)
C20	0.0346 (13)	0.0354 (13)	0.0372 (12)	0.0033 (11)	0.0070 (10)	0.0001 (10)
C21	0.0389 (13)	0.0449 (15)	0.0240 (11)	0.0047 (11)	0.0045 (10)	−0.0018 (10)
C22	0.0685 (19)	0.0431 (16)	0.0476 (15)	0.0043 (14)	0.0055 (13)	0.0095 (12)
C23	0.0322 (13)	0.0363 (13)	0.0273 (11)	0.0027 (10)	0.0076 (10)	0.0001 (9)
C24	0.0347 (13)	0.0396 (14)	0.0260 (11)	0.0013 (10)	0.0100 (10)	0.0009 (9)
C25	0.0369 (13)	0.0367 (13)	0.0262 (11)	0.0080 (10)	0.0104 (9)	0.0054 (9)
C26	0.0372 (14)	0.0653 (18)	0.0364 (13)	0.0044 (13)	0.0112 (11)	0.0117 (12)
C27	0.0465 (16)	0.087 (2)	0.0538 (17)	0.0042 (15)	0.0263 (14)	0.0180 (15)
C28	0.0637 (18)	0.074 (2)	0.0369 (14)	0.0116 (15)	0.0283 (13)	0.0134 (13)
C29	0.0552 (16)	0.0538 (16)	0.0269 (11)	0.0094 (13)	0.0148 (11)	0.0038 (11)
C30	0.0390 (13)	0.0385 (14)	0.0283 (11)	0.0065 (11)	0.0109 (10)	0.0022 (9)
C31	0.0377 (13)	0.0580 (16)	0.0263 (11)	−0.0042 (12)	0.0105 (10)	−0.0017 (10)
N1	0.0426 (13)	0.0590 (15)	0.0451 (12)	0.0159 (11)	0.0126 (10)	−0.0065 (10)
N2	0.0431 (11)	0.0376 (11)	0.0290 (9)	0.0023 (9)	0.0058 (8)	0.0027 (8)
N3	0.0371 (11)	0.0631 (14)	0.0225 (9)	−0.0068 (10)	0.0044 (8)	−0.0026 (9)
N4	0.0581 (14)	0.0577 (14)	0.0411 (12)	0.0102 (11)	0.0196 (11)	−0.0165 (10)
O1	0.0446 (12)	0.0786 (14)	0.0649 (12)	0.0107 (10)	0.0269 (9)	−0.0055 (10)
O2	0.0595 (11)	0.0454 (11)	0.0336 (8)	0.0034 (8)	0.0269 (8)	0.0051 (7)
O3	0.0732 (12)	0.0463 (11)	0.0445 (9)	0.0007 (9)	0.0344 (9)	0.0102 (8)
O4	0.0334 (9)	0.0706 (12)	0.0293 (8)	−0.0076 (9)	0.0080 (7)	0.0009 (8)

### Geometric parameters (Å, º)

**Table:**

C1—C9	1.335 (3)	C16—H16	0.9300
C1—O2	1.353 (3)	C17—C18	1.381 (5)
C1—H1	0.9300	C17—H17	0.9300
C2—O2	1.376 (3)	C18—C19	1.398 (4)
C2—C7	1.382 (3)	C18—H18	0.9300
C2—C3	1.392 (3)	C19—C20	1.372 (3)
C3—C4	1.362 (4)	C19—H19	0.9300
C3—H3	0.9300	C21—N2	1.444 (3)
C4—C5	1.392 (4)	C21—H21A	0.9700
C4—H4	0.9300	C21—H21B	0.9700
C5—C6	1.369 (3)	C22—N2	1.465 (3)
C5—H5	0.9300	C22—H22A	0.9600
C6—C7	1.403 (3)	C22—H22B	0.9600
C6—H6	0.9300	C22—H22C	0.9600
C7—C8	1.460 (3)	C23—O4	1.222 (3)
C8—O3	1.239 (3)	C23—C24	1.454 (3)
C8—C9	1.453 (3)	C24—C31	1.383 (3)
C9—C10	1.505 (3)	C24—C25	1.445 (3)
C10—C21	1.521 (3)	C25—C26	1.385 (3)
C10—C11	1.581 (3)	C25—C30	1.402 (3)
C10—H10	0.9800	C26—C27	1.383 (3)
C11—C12	1.474 (3)	C26—H26	0.9300
C11—C23	1.570 (3)	C27—C28	1.390 (4)
C11—C13	1.603 (3)	C27—H27	0.9300
C12—N1	1.138 (3)	C28—C29	1.367 (4)
C13—N2	1.454 (3)	C28—H28	0.9300
C13—C20	1.497 (3)	C29—C30	1.386 (3)
C13—C14	1.580 (3)	C29—H29	0.9300
C14—O1	1.214 (3)	C30—N3	1.377 (3)
C14—N4	1.349 (3)	C31—N3	1.343 (3)
C15—C16	1.378 (4)	C31—H31	0.9300
C15—C20	1.389 (3)	N3—H3A	0.8600
C15—N4	1.393 (3)	N4—H4A	0.8600
C16—C17	1.369 (4)		
			
C9—C1—O2	125.1 (2)	C17—C18—C19	120.3 (3)
C9—C1—H1	117.5	C17—C18—H18	119.9
O2—C1—H1	117.5	C19—C18—H18	119.9
O2—C2—C7	121.33 (19)	C20—C19—C18	118.2 (3)
O2—C2—C3	116.4 (2)	C20—C19—H19	120.9
C7—C2—C3	122.2 (2)	C18—C19—H19	120.9
C4—C3—C2	118.2 (2)	C19—C20—C15	120.1 (2)
C4—C3—H3	120.9	C19—C20—C13	130.6 (2)
C2—C3—H3	120.9	C15—C20—C13	109.3 (2)
C3—C4—C5	121.3 (2)	N2—C21—C10	103.20 (17)
C3—C4—H4	119.4	N2—C21—H21A	111.1
C5—C4—H4	119.4	C10—C21—H21A	111.1
C6—C5—C4	119.9 (2)	N2—C21—H21B	111.1
C6—C5—H5	120.0	C10—C21—H21B	111.1
C4—C5—H5	120.0	H21A—C21—H21B	109.1
C5—C6—C7	120.4 (2)	N2—C22—H22A	109.5
C5—C6—H6	119.8	N2—C22—H22B	109.5
C7—C6—H6	119.8	H22A—C22—H22B	109.5
C2—C7—C6	117.9 (2)	N2—C22—H22C	109.5
C2—C7—C8	119.8 (2)	H22A—C22—H22C	109.5
C6—C7—C8	122.3 (2)	H22B—C22—H22C	109.5
O3—C8—C9	121.4 (2)	O4—C23—C24	121.2 (2)
O3—C8—C7	122.7 (2)	O4—C23—C11	118.47 (18)
C9—C8—C7	115.85 (18)	C24—C23—C11	120.25 (19)
C1—C9—C8	119.1 (2)	C31—C24—C25	106.26 (18)
C1—C9—C10	123.3 (2)	C31—C24—C23	129.4 (2)
C8—C9—C10	117.54 (18)	C25—C24—C23	124.3 (2)
C9—C10—C21	116.64 (18)	C26—C25—C30	118.7 (2)
C9—C10—C11	113.37 (17)	C26—C25—C24	135.1 (2)
C21—C10—C11	102.75 (16)	C30—C25—C24	106.2 (2)
C9—C10—H10	107.9	C27—C26—C25	118.5 (2)
C21—C10—H10	107.9	C27—C26—H26	120.7
C11—C10—H10	107.9	C25—C26—H26	120.7
C12—C11—C23	109.35 (17)	C26—C27—C28	121.6 (3)
C12—C11—C10	110.51 (16)	C26—C27—H27	119.2
C23—C11—C10	110.54 (17)	C28—C27—H27	119.2
C12—C11—C13	110.41 (18)	C29—C28—C27	121.2 (2)
C23—C11—C13	111.63 (16)	C29—C28—H28	119.4
C10—C11—C13	104.32 (16)	C27—C28—H28	119.4
N1—C12—C11	179.7 (3)	C28—C29—C30	117.1 (2)
N2—C13—C20	113.88 (18)	C28—C29—H29	121.5
N2—C13—C14	113.76 (17)	C30—C29—H29	121.5
C20—C13—C14	101.03 (17)	N3—C30—C29	129.1 (2)
N2—C13—C11	102.17 (16)	N3—C30—C25	107.93 (19)
C20—C13—C11	116.32 (17)	C29—C30—C25	123.0 (2)
C14—C13—C11	110.14 (18)	N3—C31—C24	109.9 (2)
O1—C14—N4	126.6 (2)	N3—C31—H31	125.1
O1—C14—C13	126.2 (2)	C24—C31—H31	125.1
N4—C14—C13	107.2 (2)	C21—N2—C13	107.89 (17)
C16—C15—C20	122.2 (3)	C21—N2—C22	114.99 (19)
C16—C15—N4	127.9 (2)	C13—N2—C22	114.23 (18)
C20—C15—N4	109.9 (2)	C31—N3—C30	109.70 (19)
C17—C16—C15	117.0 (3)	C31—N3—H3A	125.2
C17—C16—H16	121.5	C30—N3—H3A	125.2
C15—C16—H16	121.5	C14—N4—C15	112.2 (2)
C16—C17—C18	122.1 (3)	C14—N4—H4A	123.9
C16—C17—H17	118.9	C15—N4—H4A	123.9
C18—C17—H17	118.9	C1—O2—C2	118.70 (17)
			
O2—C2—C3—C4	−177.9 (2)	N4—C15—C20—C19	174.1 (2)
C7—C2—C3—C4	2.2 (3)	C16—C15—C20—C13	177.2 (2)
C2—C3—C4—C5	−0.3 (4)	N4—C15—C20—C13	−4.1 (3)
C3—C4—C5—C6	−1.6 (4)	N2—C13—C20—C19	−49.2 (3)
C4—C5—C6—C7	1.6 (4)	C14—C13—C20—C19	−171.6 (3)
O2—C2—C7—C6	177.87 (19)	C11—C13—C20—C19	69.2 (3)
C3—C2—C7—C6	−2.1 (3)	N2—C13—C20—C15	128.8 (2)
O2—C2—C7—C8	−2.4 (3)	C14—C13—C20—C15	6.4 (2)
C3—C2—C7—C8	177.6 (2)	C11—C13—C20—C15	−112.8 (2)
C5—C6—C7—C2	0.2 (3)	C9—C10—C21—N2	−89.1 (2)
C5—C6—C7—C8	−179.5 (2)	C11—C10—C21—N2	35.6 (2)
C2—C7—C8—O3	−175.6 (2)	C12—C11—C23—O4	−135.8 (2)
C6—C7—C8—O3	4.1 (3)	C10—C11—C23—O4	−13.9 (3)
C2—C7—C8—C9	4.0 (3)	C13—C11—C23—O4	101.7 (2)
C6—C7—C8—C9	−176.31 (19)	C12—C11—C23—C24	47.7 (3)
O2—C1—C9—C8	0.8 (3)	C10—C11—C23—C24	169.53 (19)
O2—C1—C9—C10	−179.79 (19)	C13—C11—C23—C24	−74.8 (2)
O3—C8—C9—C1	176.4 (2)	O4—C23—C24—C31	177.3 (2)
C7—C8—C9—C1	−3.2 (3)	C11—C23—C24—C31	−6.3 (4)
O3—C8—C9—C10	−3.0 (3)	O4—C23—C24—C25	−4.6 (4)
C7—C8—C9—C10	177.35 (17)	C11—C23—C24—C25	171.8 (2)
C1—C9—C10—C21	18.8 (3)	C31—C24—C25—C26	−178.7 (3)
C8—C9—C10—C21	−161.80 (19)	C23—C24—C25—C26	2.8 (4)
C1—C9—C10—C11	−100.3 (2)	C31—C24—C25—C30	1.0 (3)
C8—C9—C10—C11	79.1 (2)	C23—C24—C25—C30	−177.5 (2)
C9—C10—C11—C12	−5.8 (3)	C30—C25—C26—C27	−0.2 (4)
C21—C10—C11—C12	−132.58 (19)	C24—C25—C26—C27	179.5 (3)
C9—C10—C11—C23	−127.01 (19)	C25—C26—C27—C28	0.9 (4)
C21—C10—C11—C23	106.2 (2)	C26—C27—C28—C29	−0.8 (5)
C9—C10—C11—C13	112.85 (19)	C27—C28—C29—C30	0.1 (4)
C21—C10—C11—C13	−13.9 (2)	C28—C29—C30—N3	−178.7 (3)
C23—C11—C12—N1	−139 (100)	C28—C29—C30—C25	0.6 (4)
C10—C11—C12—N1	99 (58)	C26—C25—C30—N3	178.9 (2)
C13—C11—C12—N1	−16 (58)	C24—C25—C30—N3	−0.9 (3)
C12—C11—C13—N2	106.58 (18)	C26—C25—C30—C29	−0.6 (4)
C23—C11—C13—N2	−131.55 (17)	C24—C25—C30—C29	179.6 (2)
C10—C11—C13—N2	−12.2 (2)	C25—C24—C31—N3	−0.7 (3)
C12—C11—C13—C20	−18.1 (2)	C23—C24—C31—N3	177.7 (2)
C23—C11—C13—C20	103.8 (2)	C10—C21—N2—C13	−47.0 (2)
C10—C11—C13—C20	−136.82 (18)	C10—C21—N2—C22	−175.8 (2)
C12—C11—C13—C14	−132.21 (18)	C20—C13—N2—C21	162.61 (19)
C23—C11—C13—C14	−10.3 (2)	C14—C13—N2—C21	−82.3 (2)
C10—C11—C13—C14	109.05 (18)	C11—C13—N2—C21	36.3 (2)
N2—C13—C14—O1	50.7 (3)	C20—C13—N2—C22	−68.2 (3)
C20—C13—C14—O1	173.2 (3)	C14—C13—N2—C22	46.9 (3)
C11—C13—C14—O1	−63.3 (3)	C11—C13—N2—C22	165.5 (2)
N2—C13—C14—N4	−129.2 (2)	C24—C31—N3—C30	0.2 (3)
C20—C13—C14—N4	−6.7 (2)	C29—C30—N3—C31	179.9 (2)
C11—C13—C14—N4	116.8 (2)	C25—C30—N3—C31	0.5 (3)
C20—C15—C16—C17	3.2 (4)	O1—C14—N4—C15	−175.1 (3)
N4—C15—C16—C17	−175.2 (3)	C13—C14—N4—C15	4.9 (3)
C15—C16—C17—C18	0.2 (5)	C16—C15—N4—C14	177.9 (3)
C16—C17—C18—C19	−2.2 (5)	C20—C15—N4—C14	−0.7 (3)
C17—C18—C19—C20	0.9 (4)	C9—C1—O2—C2	1.0 (3)
C18—C19—C20—C15	2.3 (4)	C7—C2—O2—C1	−0.1 (3)
C18—C19—C20—C13	−179.9 (2)	C3—C2—O2—C1	179.87 (19)
C16—C15—C20—C19	−4.5 (4)		

### Hydrogen-bond geometry (Å, º)

**Table:**

*D*—H···*A*	*D*—H	H···*A*	*D*···*A*	*D*—H···*A*
N3—H3*A*···O4^i^	0.86	2.14	2.967 (3)	161
N4—H4*A*···O3^ii^	0.86	2.06	2.866 (3)	156
C29—H29···O2^iii^	0.93	2.59	3.267 (3)	131

Symmetry codes: (i) *x*+1/2, −*y*+1/2, *z*+1/2; (ii) −*x*+1/2, *y*+1/2, −*z*+1/2; (iii) *x*, *y*, *z*+1.
