# Supplementary figures and images for: Crystal structure of 3′-(1H-indole-3-carbon­yl)-1′-methyl-2-oxo-4′-(4-oxo-4H-chromen-3-yl)spiro­[indoline-3,2′-pyrrolidine]-3′-carbo­nitrile
Source: Acta Crystallogr E Crystallogr Commun. 2015 Oct 31;71(Pt 11):o898–9. doi: 10.1107/S2056989015020174 (PMC4645027; doi:10.1107/S2056989015020174)

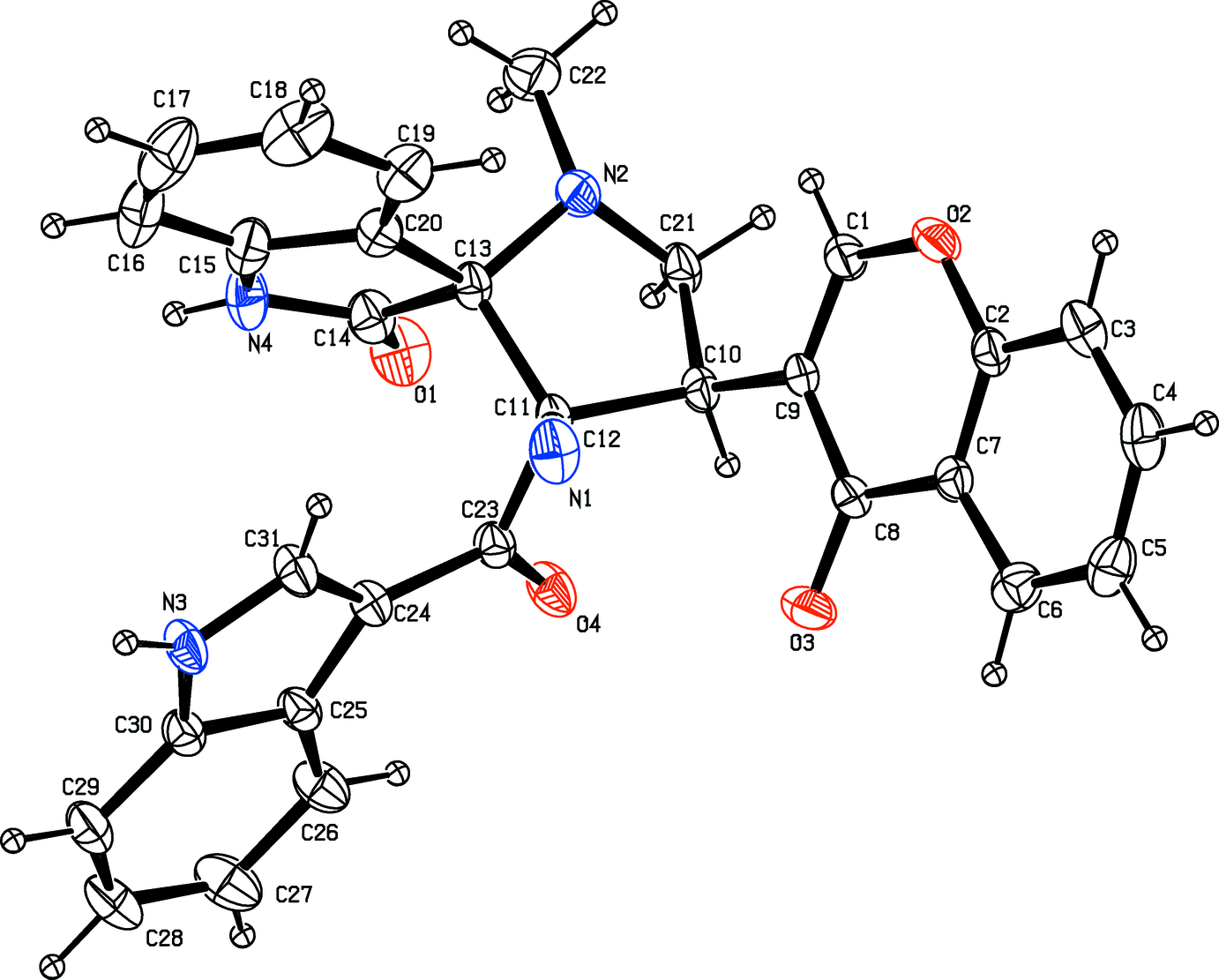

Supplement: Supplementary file 4 [file e-71-0o898-fig1.tif]

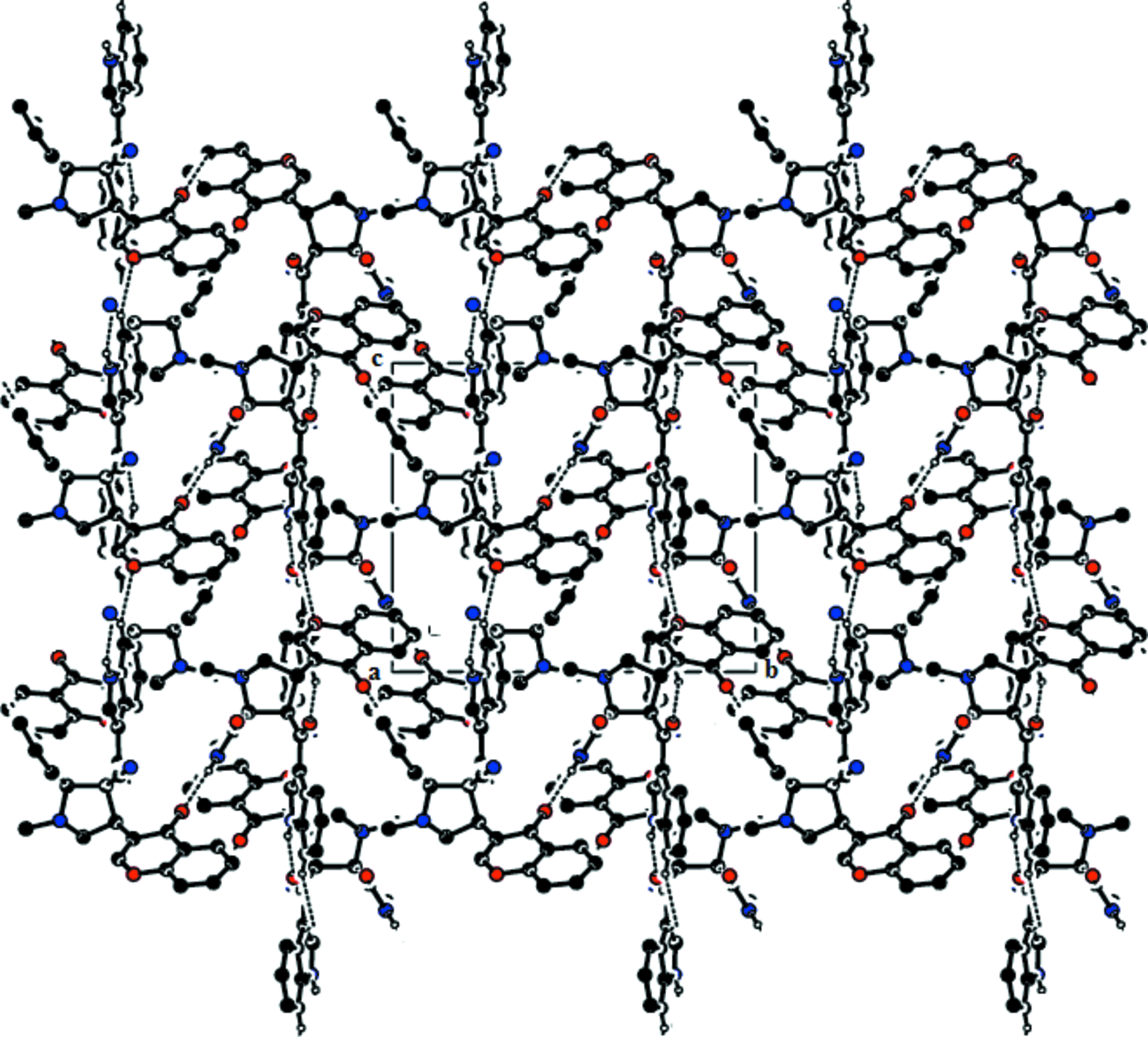

Supplement: Supplementary file 5 [file e-71-0o898-fig2.tif]
